# Effects of an orexin receptor 2-selective agonist on salivary secretion in rats

**DOI:** 10.1038/s41598-026-44080-9

**Published:** 2026-03-17

**Authors:** Jose Ichishima, Masanori Nakakariya, Haruhide Kimura

**Affiliations:** 1 Neuroscience Drug Discovery Unit, Research, Takeda Pharmaceutical Company Limited, 26-1, Muraoka-Higashi 2-Chome, Fujisawa, Kanagawa 251-8555 Japan; 2 Drug Metabolism, Pharmacokinetics and Modeling, Data and Quantitative Sciences, Takeda Pharmaceutical Company Limited, 26-1, Muraoka-Higashi 2-Chome, Fujisawa, Kanagawa 251-8555 Japan

**Keywords:** Orexin, Orexin receptor 2 (OX2R), OX2R-selective agonist, OX-202, Pilocarpine, Salivation, Medical research, Neuroscience, Physiology

## Abstract

Narcolepsy type 1 (NT1) is caused by a significant loss of orexin-producing neurons. In clinical trials, multiple orexin receptor 2 (OX2R)-selective agonists (e.g., danavorexton, TAK-994, and oveporexton) improved wakefulness and decreased cataplexy in individuals with NT1. However, OX2R-selective agonists were also reported to induce hypersalivation as an adverse event in a small number of healthy volunteers and in individuals with NT1. Importantly, no objective data supporting these observations are available, and multiple factors can indirectly affect saliva secretion. In this study, we assessed the effect of an OX2R-selective agonist, OX-202, on salivary secretion in anesthetized or freely moving rats using a muscarinic acetylcholine receptor agonist, pilocarpine, as a positive control. Subcutaneous administration of pilocarpine at 1 mg/kg significantly increased the amount of saliva in the oral cavity in both anesthetized and freely moving rats. In contrast, intraperitoneal administration of OX-202 at 100 mg/kg under anesthetized conditions did not increase salivary secretion in rats. Moreover, oral administration of OX-202 (30 and 100 mg/kg) at zeitgeber time 6 (sleep phase) or zeitgeber time 15 (active phase) did not increase salivary secretion in the oral cavity in freely moving rats. In conclusion, OX2R-selective agonists may not directly induce salivary secretion in rats.

## Introduction

Orexin peptides, orexin A (OX-A) and orexin B, are produced in orexin neurons in the lateral hypothalamus (LH)^1,2^. OX-A has similar agonistic activity to orexin receptor 1 (OX1R) and orexin receptor 2 (OX2R), while orexin B shows moderate selectivity for OX2R over OX1R^2,3^. Narcolepsy type 1 (NT1) is a chronic, rare, neurological disorder characterized by excessive daytime sleepiness and cataplexy, and is associated with the loss of orexin neurons^4,5^. OX2R plays a pivotal role in NT1 symptoms; thus, multiple OX2R-selective agonists are currently in development as novel therapeutic drugs for NT1^6–8^. To date, OX2R-selective agonists (e.g., danavorexton, TAK-994, and oveporexton) have improved excessive daytime sleepiness and reduced cataplexy in individuals with NT1^6–8^. In clinical studies, hypersalivation was reported as an adverse event in a small number of healthy volunteers and individuals with narcolepsy receiving OX2R-selective agonists^6–8^. However, no objective approach was used to confirm these observations.

Saliva is produced by three major salivary glands, including parotid glands, submandibular glands, and sublingual glands^9^, and its secretion is modulated by the autonomic (sympathetic and parasympathetic) nervous systems and other factors such as taste stimulation, aging, diseases, and medication intake, resulting in large individual differences in humans^10,11^. Several studies suggest the LH is involved in the neural regulation of salivary secretion^12–14^. For example, retrograde tracing has demonstrated that LH nucleus neurons directly project to the superior salivatory nucleus in rats^12^. However, the role of the orexin system in saliva secretion has not been verified.

In this study, we assessed the effects of a tool OX2R-selective agonist, OX-202, on salivary secretion in rats. Rats represent a well-established model for evaluating various functions of OX2R-selective agonists, including wake-promoting effects^15–17^, and for the salivary secretion induced by various sialagogues (e.g., pilocarpine and cevimeline)^18–20^. Therefore, we used rats to assess the effect of OX-202 on salivary secretion. Saliva measurement under anesthesia can prevent saliva swallowing. On the other hand, anesthesia can suppress neuronal activities in the brain^21^, resulting in the reduction of salivary secretion. Therefore, we assessed salivary secretion in both anesthetized and freely moving rats. Furthermore, diurnal fluctuation of OX-A with higher levels during the active phase and lower levels during the sleep phase may influence OX2R-selective agonist–induced salivary secretion. Thus, we examined the effects of OX-202 on salivary secretion during both the sleep and active phases in freely moving rats^22,23^.

## Results

### Effect of OX-202 on saliva secretion in anesthetized rats

**Fig. 1:**
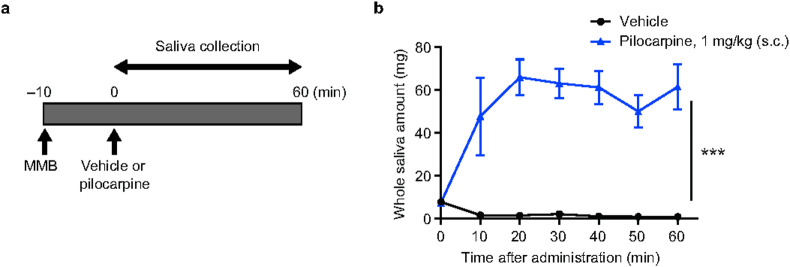
Effects of pilocarpine on salivary secretion in anesthetized rats. (**a**) Time schedule for saliva collection in anesthetized rats. Pilocarpine was administered s.c. at 10 min after medetomidine, midazolam, and butorphanol (MMB)-induced anesthesia. Saliva collection was performed for 60 min after administration. (**b**) Effects of pilocarpine at 1 mg/kg (s.c.) on salivary secretion in anesthetized rats. Mean ± standard error of the mean; *n* = 4. Repeated measures analysis of variance; ***P < 0.001.

We established experimental conditions to measure salivary secretion using pilocarpine, a muscarinic acetylcholine receptor agonist, as a positive control in anesthetized rats. Rats were anesthetized with medetomidine, midazolam, and butorphanol, and remained under anesthesia for more than 60 min. Vehicle or pilocarpine (1 mg/kg) was subcutaneously (s.c.) administered to rats at 10 min after anesthesia, and then the amount of saliva in the oral cavity was measured for 60 min (Fig. 1a). The vehicle-treated rats showed a time-dependent decrease in the amount of saliva, perhaps due to the effects of anesthesia. On the contrary, pilocarpine (1 mg/kg) significantly increased the amount of saliva in anesthetized rats (Fig. 1b).

**Fig. 2:**
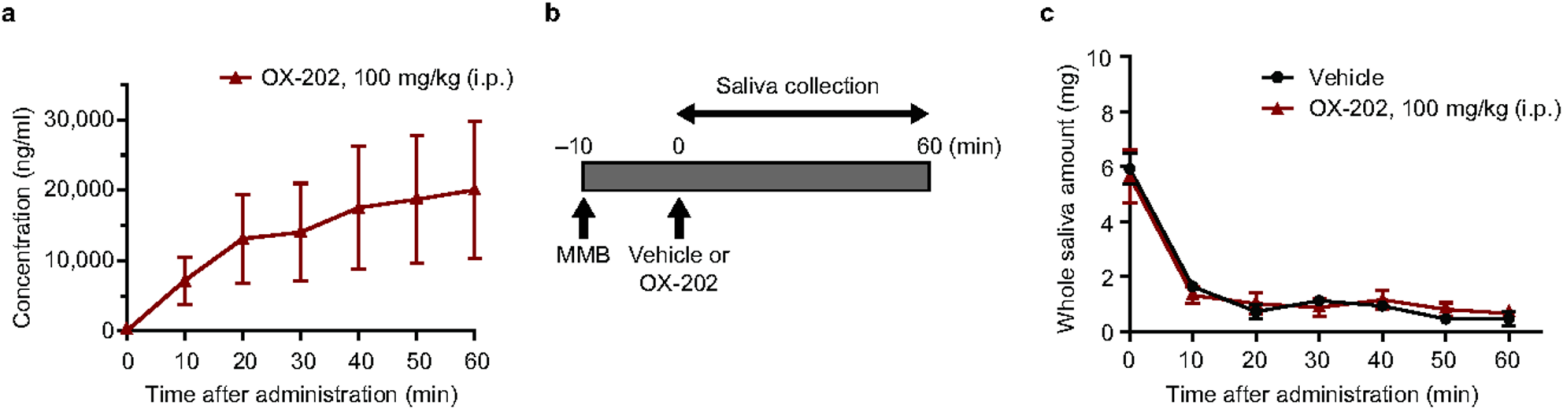
Effects of OX-202 on salivary secretion in anesthetized rats. (**a**) Plasma concentration of OX-202 (100 mg/kg) after i.p. administration in anesthetized rats. Mean ± standard error of the mean; *n* = 3. (**b**) Time schedule for saliva collection in anesthetized rats. OX-202 was administered i.p. at 10 min after medetomidine, midazolam, and butorphanol (MMB)-induced anesthesia. Saliva collection was performed for 60 min after administration. (**c**) Effects of OX-202 at 100 mg/kg (i.p.) on salivary secretion in anesthetized rats. Mean ± standard error of the mean; *n* = 6–7.

Next, we characterized the effects of an OX2R-selective agonist, OX-202, on salivation under the same conditions. OX-202 activated OX2R with a 50% effective concentration (EC_50_) value of 6.9 nM with weak OX1R agonistic activity (EC_50_ value of 2,900 nM)^16^. OX2R-selective agonist, OX-201, promotes wakefulness through activation of the central nervous system, thus wake-promoting effects are a good pharmacodynamic marker for high brain exposure of an OX2R agonist^24^. In our previous study, oral (p.o.) administration of OX-202 at 30 mg/kg induced potent wake-promoting effects, with a threshold plasma concentration (awake > 75% time) of approximately 3,000 ng/ml during the sleep phase^16^. Under anesthetized conditions, p.o. administration is not technically feasible; therefore, we explored conditions to achieve the plasma concentration of > 3,000 ng/mL by intraperitoneal (i.p.) administration. We found that the plasma concentration of OX-202 exceeded 3,000 ng/mL within 5 min after i.p. administration at 100 mg/kg (Fig. 2a). Next, the effects of OX-202 at 100 mg/kg (i.p.) on saliva secretion under anesthesia were examined (Fig. 2b). Anesthesia time-dependently reduced the amount of saliva in the vehicle-treated rats, and OX-202 (100 mg/kg i.p.) did not affect salivary secretion compared with vehicle-treated rats (Fig. 2c). Thus, OX-202 at wake-promoting plasma concentrations did not induce salivary secretion in anesthetized rats.

### Effect of OX-202 on saliva secretion during the sleep phase in freely moving rats

**Fig. 3:**
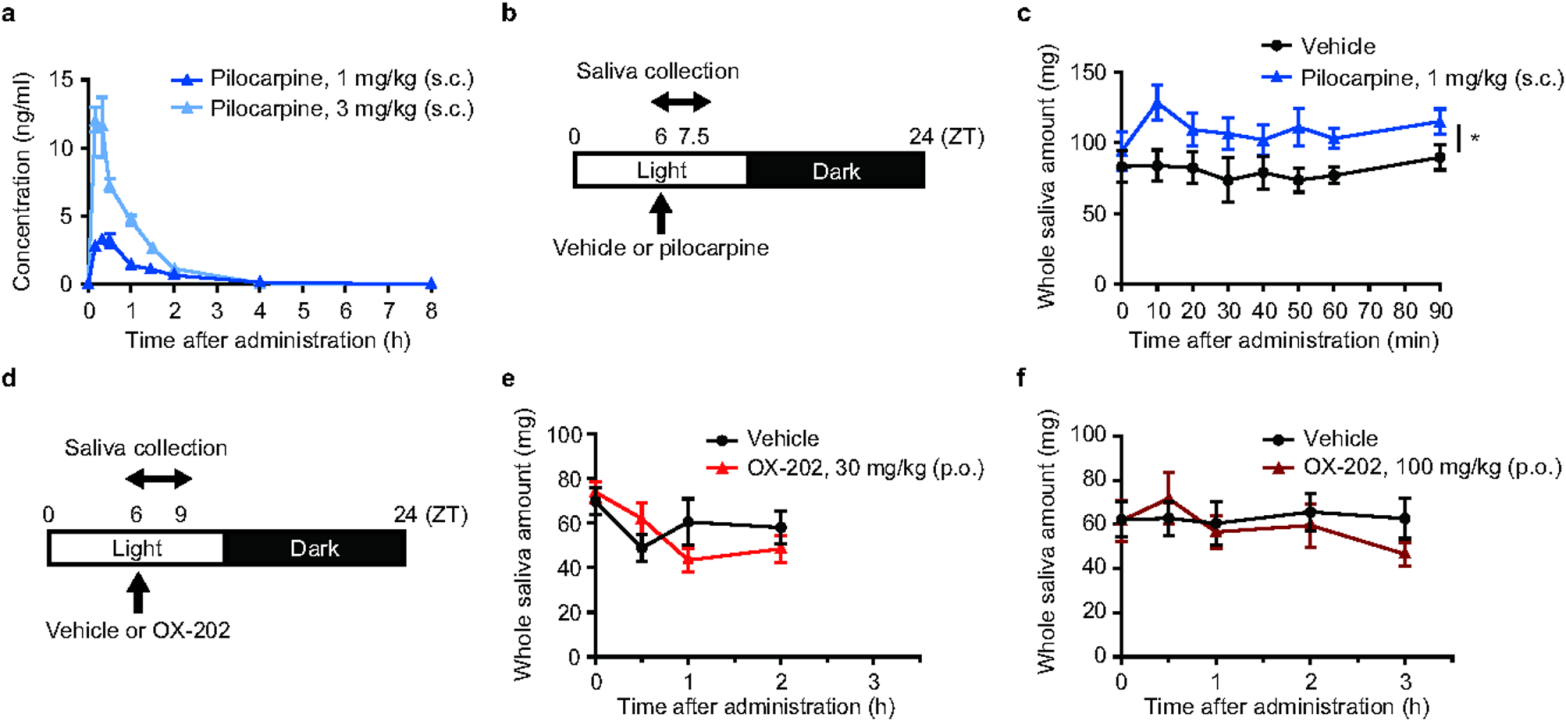
Effects of pilocarpine and OX-202 on salivary secretion in freely moving rats during the sleep phase. (**a**) Plasma concentrations of pilocarpine at 1 or 3 mg/kg after s.c. administration in freely moving rats. Mean ± standard error of the mean (s.e.m.); *n* = 3. (**b**) Time schedule for saliva collection in freely moving rats during the sleep phase following s.c. administration of pilocarpine at ZT6. Saliva collection was performed for 90 min after administration. (**c**) Effects of pilocarpine at 1 mg/kg (s.c.) on salivary secretion in freely moving rats during the sleep phase. Mean ± s.e.m.; *n* = 8; *P < 0.05. (**d**) Time schedule for saliva collection in freely moving rats during the sleep phase following p.o. administration of OX-202 at ZT6. Saliva collection was performed for up to 2 (OX-202, 30 mg/kg) or 3 (OX-202, 100 mg/kg) h after administration. Effects of OX-202 at (**e**) 30 mg/kg (p.o.) and (**f**) 100 mg/kg (p.o.) on salivary secretion in freely moving rats during the sleep phase. Mean ± s.e.m.; *n* = 8.

During the sleep phase, pilocarpine was administered to rats and its pharmacokinetic profile investigated. After injection of pilocarpine at 1 and 3 mg/kg (s.c.), the time to maximum concentration (T_max_) was approximately 20 min (Fig. 3a). Pilocarpine at 1 mg/kg was administered s.c. to freely moving rats at zeitgeber time (ZT) 6. Then saliva was collected at various time points (10, 20, 30, 40, 50, 60, and 90 min) up to 90 min after administration (Fig. 3b), because pilocarpine plasma concentration at 2 h after administration was very low (Fig. 3a). Pilocarpine (1 mg/kg s.c.) significantly increased salivary secretion during the sleep phase, with the highest level at approximately 10 min after administration in freely moving rats (Fig. 3c).

Under the experimental conditions, the effect of p.o. administration of OX-202 on salivary secretion was investigated. In our previous study, p.o. administration of OX-202 at 30 and 100 mg/kg showed T_max_ values of 1.8 h and 2.6 h, respectively, and significantly increased wakefulness time for 4 h and ≥ 6 h, respectively, after administration in rats^16^. Therefore, OX-202 was administered p.o. to freely moving rats at ZT6 (sleep phase), then saliva was collected by the time that its plasma exposure reached the highest level (up to 2 h for 30 mg/kg and 3 h for 100 mg/kg) (Fig. 3d). OX-202 (30 and 100 mg/kg p.o.) did not increase the amount of saliva during the sleep phase in freely moving rats (Fig. 3e and f).

### Effect of OX-202 on saliva secretion during the active phase in freely moving rats

**Fig. 4:**
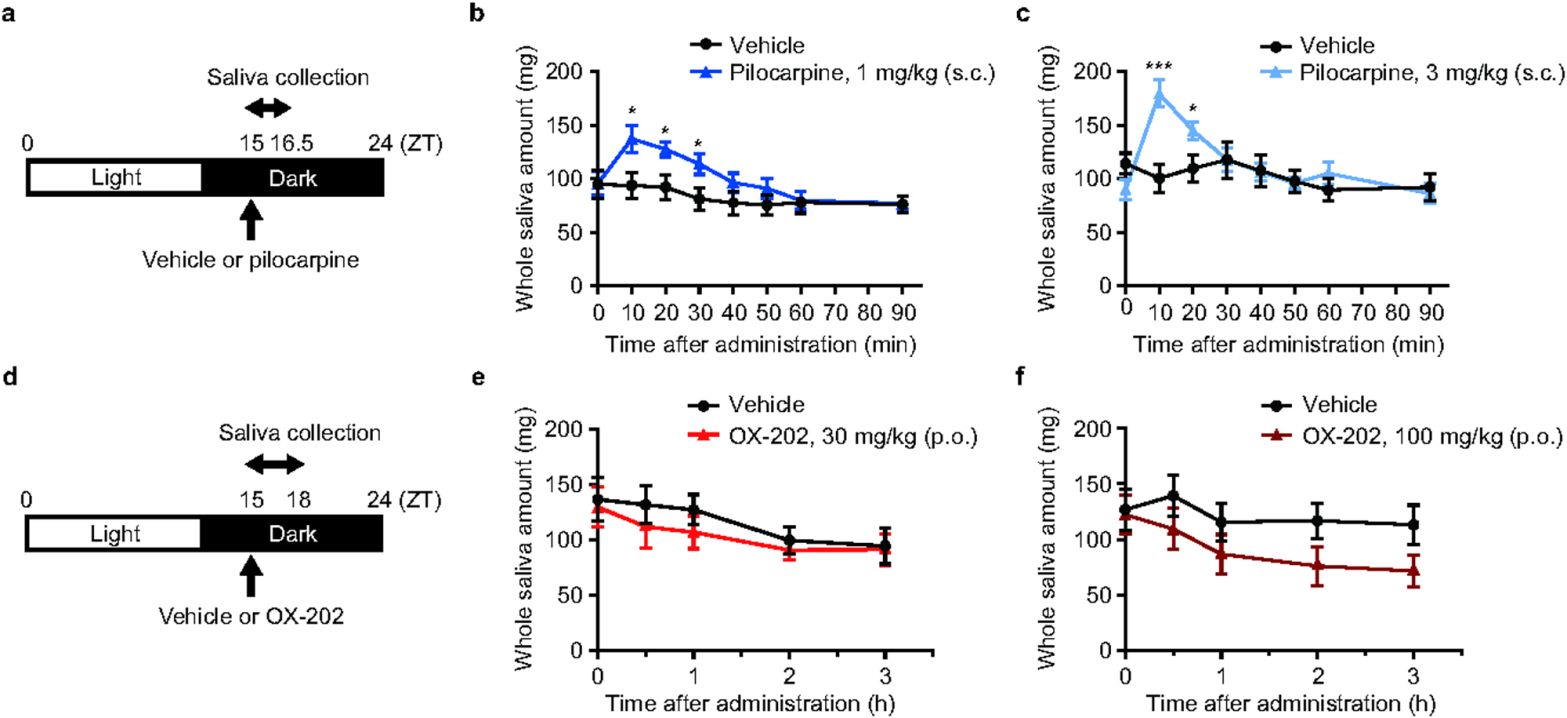
Effects of pilocarpine and OX-202 on salivary secretion in freely moving rats during the active phase. (**a**) Time schedule for saliva collection in freely moving rats during the active phase following s.c. administration of pilocarpine at ZT15. Saliva collection was performed for 1.5 h after administration. Effects of pilocarpine at (**b**) 1 mg/kg (s.c.) and (**c**) 3 mg/kg (s.c.) on salivary secretion in freely moving rats during the active phase. Mean ± s.e.m.; *n* = 8. Repeated measures analysis of variance followed by post hoc Student’s *t*-test, if the interaction between time and group was significant; *P < 0.05, ***P < 0.001. (**d**) Time schedule for saliva collection in freely moving rats during the active phase following p.o. administration of OX-202 at ZT15. Saliva collection was performed for 3 h after administration. Effects of OX-202 at (**e**) 30 mg/kg (p.o.) and (**f**) 100 mg/kg (p.o.) on salivary secretion in freely moving rats during the active phase. Mean ± s.e.m.; *n* = 8.

Next, salivation was examined in the active phase, when OX-A levels are high^23^. Pilocarpine was administered s.c. to freely moving rats at ZT15, then saliva was collected for 1.5 h (Fig. 4a). Pilocarpine (1 and 3 mg/kg s.c.) rapidly increased the amount of saliva at 10 min after administration, with the effect declining after 30–40 min, consistent with the pharmacokinetic data (Fig. 4b and c).

Then, OX-202 at 30 and 100 mg/kg was administered p.o. to freely moving rats at ZT15, and the amount of saliva in the oral cavity was measured for 3 h (Fig. 4d). OX-202 (30 and 100 mg/kg p.o.) did not increase the amount of saliva in freely moving rats (Fig. 4e and f). Conversely, a slight decrease in salivation was observed in both treatment groups, though the change was not statistically significant.

## Discussion

In this study, pilocarpine, used as a positive control, significantly increased saliva secretion in both anesthetized and freely moving rats. Under the same conditions, OX-202, an OX2R-selective agonist, did not increase salivary secretion in rats. It was hypothesized that the promotion of wakefulness might induce salivary secretion, as salivary secretion is lower during the sleep phase versus wakefulness states^25^. In fact, the average amount of saliva collected at the 0 min time point across the vehicle-, pilocarpine-, or OX-202-treated groups of these studies ranged from 89–137 mg in the active phase and 61–95 mg in the sleep phase. By using our original method to accurately measure orexin-A, we previously showed that orexin levels were also low during the sleep phase in monkey cerebrospinal fluid (CSF)^26^. Together with clinical reports for hypersalivation as an adverse event of OX2R agonists, orexin may be involved in the regulation of salivation. However, in this study, no significant increase in salivation was observed, even at potent wake-promoting doses of OX-202 (30 and 100 mg/kg p.o.) in both the sleep and active phases.

There is a report that OX2R is expressed on orexin neurons, suggesting the OX2R agonist-induced release of orexin via activation of orexin neurons^27^. However, we previously showed that OX2R agonist-induced wakefulness does not modify the orexin-A level in monkey CSF^28^. In the sleep phase study, as the blood concentration of OX-202 exceeded the threshold plasma concentration for potent wakefulness (3,000 ng/mL), OX-202–treated rats should be awake besides at the zero time point. Therefore, this study was done under conditions of wake-promoting OX-202 plasma concentrations and low/nighttime orexin-A levels in awake rats.

Orexin neurons project throughout the whole brain and exert diverse functions^29^. In fact, OX2R protein is widely expressed across the brain in rodents^30^. There is a report showing OX2R expression in the rat superior salivatory nucleus, which regulates salivation from salivary glands^31^. However, our data indicate that OX-202 does not directly increase salivation in rats under various conditions. Protein expression does not always correspond to functional activity; therefore, characterization of a receptor function with highly selective small-molecule ligands is critically important^15,24,32^.

In clinical trials with danavorexton, TAK-994, and oveporexton, salivary hypersecretion was reported in 5–20% of treated participants versus 0–5% from placebo-treated participants^7,8^. Therefore, there may be a discrepancy between preclinical findings and clinical observations of salivary secretion. Clinical studies rely on participants self-reporting adverse events, such as experiencing salivary secretion, but information regarding the intensity of these secretions was not collected. Thus, the magnitude of the effects of an OX2R agonist on salivary secretion in humans is unclear. It is also important to note that various factors, such as activities of the autonomic nervous system, taste sensation, aging, and medication intake, can affect the regulation of salivation, resulting in large individual differences in humans^11^. As OX2R activation can modulate functions of the various neuronal pathways, including the autonomic nervous system, OX2R agonists may indirectly promote salivation in humans.

OX2R activation was previously considered as an undruggable target; however, due to recent breakthroughs^6–8^, multiple OX2R-selective agonists are in development^6–8^. Notably, oveporexton achieved positive results in Phase 3 clinical trials^33^. This preclinical study can provide limited insights into the mechanisms underlying saliva secretion. However, considering the promising properties of OX2R-selective agonists as first-in-class drugs for multiple diseases, detailed characterization of the function of OX2R agonists are highly valuable for the medical field and in turn patients.

In conclusion, OX-202 may not directly induce salivary secretion in either anesthetized or freely moving rats. As saliva secretion can be sensitized by multiple factors, lack of studies to assess if OX2R agonists may indirectly promote salivation induced by various environmental stimuli is a limitation of this work.

## Methods

### Animals

Male Sprague–Dawley rats (The Jackson Laboratory Japan, Kanagawa, Japan) were housed under laboratory conditions (12-h light/dark cycles), given water ad libitum, and fed a commercial diet. In this study, 65 rats were used in total, and their use in each experiment is described as follows: saliva collection was performed in 24 rats (8–10 weeks old, 330–600 g, separate batches of 8 rats were used for each compound dose) during the sleep phase, 32 rats (10–14 weeks old, 360–570 g, separate batches of 8 rats were used for each compound dose) during the active phase, and 21 rats (8–10 weeks old, 280–530 g, 8 rats were used for pilocarpine and 13 rats were used for OX-202) under anesthesia. Pharmacokinetic studies were conducted in 9 rats (8–13 weeks old, 260–530 g, separate batches of 3 rats were used for each compound dose). At the end of the study, rats were euthanized by carbon dioxide (CO_2_) inhalation using a gradual-fill method at a displacement rate of 50% of the chamber volume per minute. Following CO_2_ exposure, death was confirmed by the irreversible absence of respiratory movements.

The care and use of the animals and the experimental protocols used in this study were approved by the Institutional Animal Care and Use Committee of Shonan Health Innovation Park (Kanagawa, Japan; approval ID: AU-00040329). General procedures for animal care and housing were in accordance with the current AAALAC recommendations. All animal experiments were performed in accordance with ARRIVE guidelines.

### Chemicals and solutions

OX-202 was synthesized by Takeda Pharmaceutical Company Limited (Fujisawa, Japan)^16^. For in vivo studies of freely moving rats, OX-202 was suspended in 0.5% (w/v) methylcellulose in distilled water (Fujifilm Wako Pure Chemical Corporation, Osaka, Japan), then OX-202 was administered p.o. to rats in a volume of 5 ml/kg of body weight. For in vivo studies of anesthetized rats, OX-202 was dissolved in 10% dimethyl sulfoxide (Fujifilm Wako Pure Chemical Corporation), 10% Kolliphor® EL (Sigma-Aldrich, St Quentin Fallavier, France), 20% Macrogol 400 (Maruishi Pharmaceutical Company Limited, Osaka, Japan), and 60% Otsuka Distilled Water (Otsuka Pharmaceutical Factory, Inc., Tokushima, Japan), then OX-202 was administered i.p. to rats in a volume of 5 ml/kg of body weight. The positive control pilocarpine hydrochloride was purchased from Selleck Chemicals (#S4231, Houston, TX, USA). Pilocarpine hydrochloride was dissolved in Otsuka Normal Saline (Otsuka Pharmaceutical Factory, Inc.), then pilocarpine was administered s.c. to rats in a volume of 5 ml/kg of body weight.

### Anesthesia

Medetomidine (0.15 mg/kg; Nippon Zenyaku Kogyo Company Limited, Fukushima, Japan), midazolam (2 mg/kg; Sandoz Kabushiki-Kaisha, Tokyo, Japan), and butorphanol (2.5 mg/kg; Meiji Animal Health Company Limited, Tokyo, Japan) were dissolved in Otsuka Normal Saline (Otsuka Pharmaceutical Factory, Inc.) and administered s.c. to rats in a volume of 2.5 ml/kg of body weight to induce anesthesia. Before compound administration, anesthesia depth was assessed by reflex responses to paw pinches. Atipamezole (0.75 mg/kg, Kyoritsu Seiyaku Corporation, Tokyo, Japan) was administered s.c. to rats in a volume 2.5 ml/kg of body weight after completion of saliva collection procedure to enhance recovery from the sedated state.

### Saliva collection

Saliva collection was performed as previously described, with some modifications^34,35^. Briefly, rats were restricted from intake of food and water for 2 h prior to saliva collection. In freely moving rats, a rat was held by hand without anesthesia and then saliva was collected following drug administration. Under anesthetized conditions, the depth of anesthesia was confirmed by reflex responses to paw pinches, and saliva collection began 10 min after anesthesia induction. Sterilized cotton swabs (Osaki Medical, Aichi, Japan) were used to wipe the oral cavity for 30 s (two sets of 15 s) to collect the whole amount of saliva produced from the rats. The amount of saliva was calculated from the change in the mass of the cotton swab. Under anesthetized conditions, swabs could strip off small pieces of cotton, due to wiping dry mouths, resulting in negative values for the change of the cotton swab mass. To avoid underestimation of the amount of saliva, negative values were assumed as zero.

### Pharmacokinetic studies of pilocarpine hydrochloride and OX-202

Blood samples were collected from the tail vein of rats at 10, 20, 30, 60, 90, 120, 240, and 480 min after administration of pilocarpine hydrochloride (1 and 3 mg/kg s.c.). Blood samples at 10, 20, 30, 40, 50, and 60 min after administration of OX-202 (100 mg/kg i.p.) were collected from anesthetized rats. Plasma was separated from the blood samples by centrifugation. The drug concentrations in plasma were quantified with high-performance liquid chromatography-tandem mass spectrometry (LC–MS/MS). Plasma samples were mixed with acetonitrile containing diclofenac as an internal standard then centrifuged. The supernatants were diluted with an appropriate volume of 10 mmol/L acetic ammonium or 0.2% (v/v) formic acid in 10 mmol/L ammonium formate and analyzed using LC–MS/MS. LC–MS/MS analysis was conducted with API5000 for pilocarpine and QTRAP 5500 for OX-202 (Applied Biosystems, Foster City, CA), respectively coupled with a turbo ion spray interface in positive ion mode and connected with Ultra Fast Liquid Chromatography (Shimadzu, Kyoto, Japan). Reverse phase chromatography (mobile phase A, 10 mmol/L acetic ammonium solution for pilocarpine or 0.2% (v/v) formic acid in 10 mM ammonium formate for OX-202; mobile phase B, acetonitrile) was used to elute and separate each drug with a InertSustain AQ-C18, C18 column (2.1 × 50 mm, 3 μm; GL Sciences, Tokyo, Japan) for pilocarpine and Shim-pack XR-ODS, C18 column (2.0 × 30 mm, 2.2 μm; Shimadzu) for OX-202, respectively. Injections of 2–5 μL were analyzed using a flow rate of 0.7 mL/min. The following transitions (precursor ion m/z > product ion m/z) were monitored: 209.04 > 68.03 for pilocarpine, 485.17 > 399.14 for OX-202, and 296.1 > 214.2 for diclofenac. The lower limit of quantitation was 0.3 ng/ml for pilocarpine and 3 ng/ml for OX-202.

### Statistical analysis

Data are presented as mean ± standard error of the mean. Statistical analysis was performed using EXSUS (SAS version 9.4, EP Croit Corporation Limited, Tokyo, Japan). To compare time-elapsed data (i.e., time course of amount of saliva), data were analyzed with repeated measures analysis of variance from the first collection time point after drug administration. If the interaction between time and group was significant, a post hoc Student’s *t*-test was followed. For all analyses, a *P* value of ≤ 0.05 was considered significant.

## Data Availability

The datasets and materials supporting the findings of this study are available from the corresponding author on reasonable request.

## Abbreviations

CSF: Cerebrospinal fluid
EC_50_: 50% Effective concentration
i.p.: Intraperitoneal
LH: Lateral hypothalamus
MMB: Medetomidine, midazolam, and butorphanol
NT1: Narcolepsy type 1
OX-A: Orexin A
OX1R: Orexin receptor 1
OXR2: Orexin receptor 2
p.o.: Oral
s.c.: Subcutaneous
s.e.m.: Standard error of the mean
T_max_: Time to maximum concentration
ZT: Zeitgeber time
